# A perspective of fluorescence microscopy for cellular structural biology with EGFR as witness

**DOI:** 10.1111/jmi.13151

**Published:** 2022-11-04

**Authors:** M. L. Martin‐Fernandez

**Affiliations:** ^1^ Central Laser Facility Science and Technology Facilities Council, Rutherford Appleton Laboratory Didcot UK

**Keywords:** EGFR, FLIM‐FRET, microscopy, single molecule, super‐resolution

## Abstract

The epidermal growth factor receptor (EGFR) is a poster child for the understanding of receptor behaviour, and of paramount importance to cell function and human health. Cloned almost forty years ago, the interest in EGFR's structure/function relationships remains unabated, not least because changes in oncogenic EGFR mutants are key drivers of the formation of lung and brain tumours. The structure of the assemblies formed by EGFR have been comprehensibly investigated by techniques such as high‐resolution X‐ray crystallography, NMR and all‐atom molecular dynamics (MD) simulations. However, the complexity embedded in the portfolio of EGFR states that are only possible in the physiological environment of cells has often proved refractory to cell‐free structural methods. Conversely, some key inroads made by quantitative fluorescence microscopy and super‐resolution have depended on exploiting the wealth of structures available. Here, a brief personal perspective is provided on how quantitative fluorescence microscopy and super‐resolution methods have cross‐fertilised with cell‐free‐derived EGFR structural information. I primarily discuss areas in which my research group has made a contribution to fill gaps in EGFR's cellular structural biology and towards developing new tools to investigate macromolecular assemblies in cells.

## 1 INTRODUCTION

Understanding the mechanisms by which cell surface receptors like EGFR transduce their cognate extracellular stimuli across the plasma membrane is an intricate challenge that can be stratified into three levels. One is to describe the conformational states that functional receptor complexes can adopt. A second is to elucidate the principles that rule the assembly and disassembly of the different states. A third is to understand supra‐molecular interactions, for example, with lipids and/or membrane proteins that regulate their dependence on the cell context. Different flavours of fluorescence microscopy are specialised to tackle the different challenges posed by each endeavour.

States adopted by signalling plasma membrane receptors, like the EGFR, can typically include monomers, which tend to be structurally and diffusionally more dynamic,^1^, ^2^ dimers,^3^ larger oligomers characterised by stoichiometry, size, geometry, and conformation,^4^, ^5^ and larger clusters, possibly underpinned by supra‐molecular interactions, and which are experimentally characterised by the type and number of receptors, their shape and size, and can vary between normal and cancer cells.^6^

The assembly and disassembly of non‐monomer states encode the information to carry out the portfolio of different signalling functions the membrane receptor is responsible for. These can include (i) receiving the incoming extracellular signal by cognate ligand binding; (ii) decoding the signal by adopting a specific receptor ectodomain conformation; (iii) transducing with specificity and fidelity the signal to the cell interior via specific allosteric conformational changes; and (iv) amplifying, modulating, thresholding, and down‐regulating the signal effects by adopting different assembly architectures, shapes, conformations and sizes that afford, for example, specific regulation of cooperativity (for a review, see, e.g., Ref. 7).

Monomer, dimer and oligomer states can orchestrate the recruitment of location‐specific adaptors and effectors and by these interactions regulate the assembly of supra‐molecular states that regulate the specificity of the signals effected at the different locations.^8^ Signalling from supra‐molecular states can also help to shape areas of the lipid bilayer with different lipid composition like lipid rafts,^9^ or regions of the plasma membrane bent by being enriched by different proteins, like coated and uncoated pits,^10^ caveolae,^11^ ruffles,^12^ filopodia,^13^ lamellipodia^14^ etc.

Fluorescence microscopy techniques are well matched to imaging molecules in specific cell locations, and are therefore regularly employed to study transmembrane receptors such as the EGFR. Popular techniques include widefield epifluorescence microscopy that provides information in the *x*, *y* plane at optical resolution (see e.g. Ref. 15) and scanning confocal microscopy, in which optical sectioning along the z‐axis is achieved by focussing the illumination and detection optics on the same diffraction‐limited spot in the sample, which is scanned in the x, y plane at different z depths in the sample, allowing the reconstruction of 3D volumes (see e.g. Ref. 16).

To ascertain properties like the size of receptor clusters, the number of interacting receptors, and/or the receptor diffusion parameters, widefield and confocal microscopy have been combined with other methods, such as fluorescence recovery after photobleaching (FRAP),^17^ imaging correlation microscopy (ICM),^18^ number and brightness (N&B) analysis^19^ and fluorescence resonance energy transfer (FRET).^20^

A popular method of exciting fluorescence from cell surface receptors is total internal reflection fluorescence (TIRF) illumination,^21^ which generates an *x*, *y* field between the upper layer of the glass sample dish and the sample buffer whose amplitude decays exponentially in the *z* direction, decaying over ∼100 nm within the sample and thus targeting most of the widefield illumination to the basal plasma membrane (for a short review see e.g. Ref. 22). Because of its increased surface‐to‐volume contrast, TIRF microscopy has often been exploited in single molecule‐type applications involving, for example, single particle tracking (SPT),^23^ single particle location‐based super‐resolution microscopy, like direct stochastic optical reconstruction microscopy dSTORM,^24^ fluorophore localisation imaging with photobleaching (FLImP)^5^ and single molecule FRET (smFRET).^25^

The above fluorescence microscopy methods investigate the state and behaviour of specific molecules in cells by detecting light from the probes that label these molecules. To help interpret results, and to infer structural information, fluorescence microscopy benefits from a partnership with cell‐free structural biology methods and molecular dynamics (MD) simulations. Using EGFR as an example, I discuss below examples of how these fields have cross‐fertilised to derive atom‐resolution structures of oligomers at the plasma membrane not amenable to be investigated by cell‐free methods.

## 2 A BRIEF FOREWORD ON EGFR

EGFR is the archetype of single‐pass membrane‐spanning receptors and one of the best‐studied signal transduction molecules.^26^ When embedded in the plasma membrane, its role is to bind cognate extracellular soluble growth factors and transduce their specific growth signals from the extracellular milieu into the cell interior.^27^ EGFR is also the founding member of the growth factor receptor tyrosine kinase super‐family, which comprises 18 sub‐groups of cell surface receptors for many growth factors, cytokines and hormones.^28^

Its significance as a key regulator of cellular growth, survival, proliferation and differentiation has engendered a wealth of multidisciplinary data on EGFR behaviour from biochemical, biophysical, imaging, genomics, proteomics, systems biology bioinformatics and computational methods.^29^ Data available include the EGFR interactome,^30^ how EGFR adaptors and downstream effector pathways relay information outputs^28^ and how spatiotemporal control of EGFR signals can be achieved by receptor trafficking through the cell endomembrane system of the endosomal network.^31^ This network is an intracellular nexus for orchestrating the trafficking of not just EGFR, but also many of the 5000+ integral membrane proteins encoded by the human genome.

**FIGURE 1:**
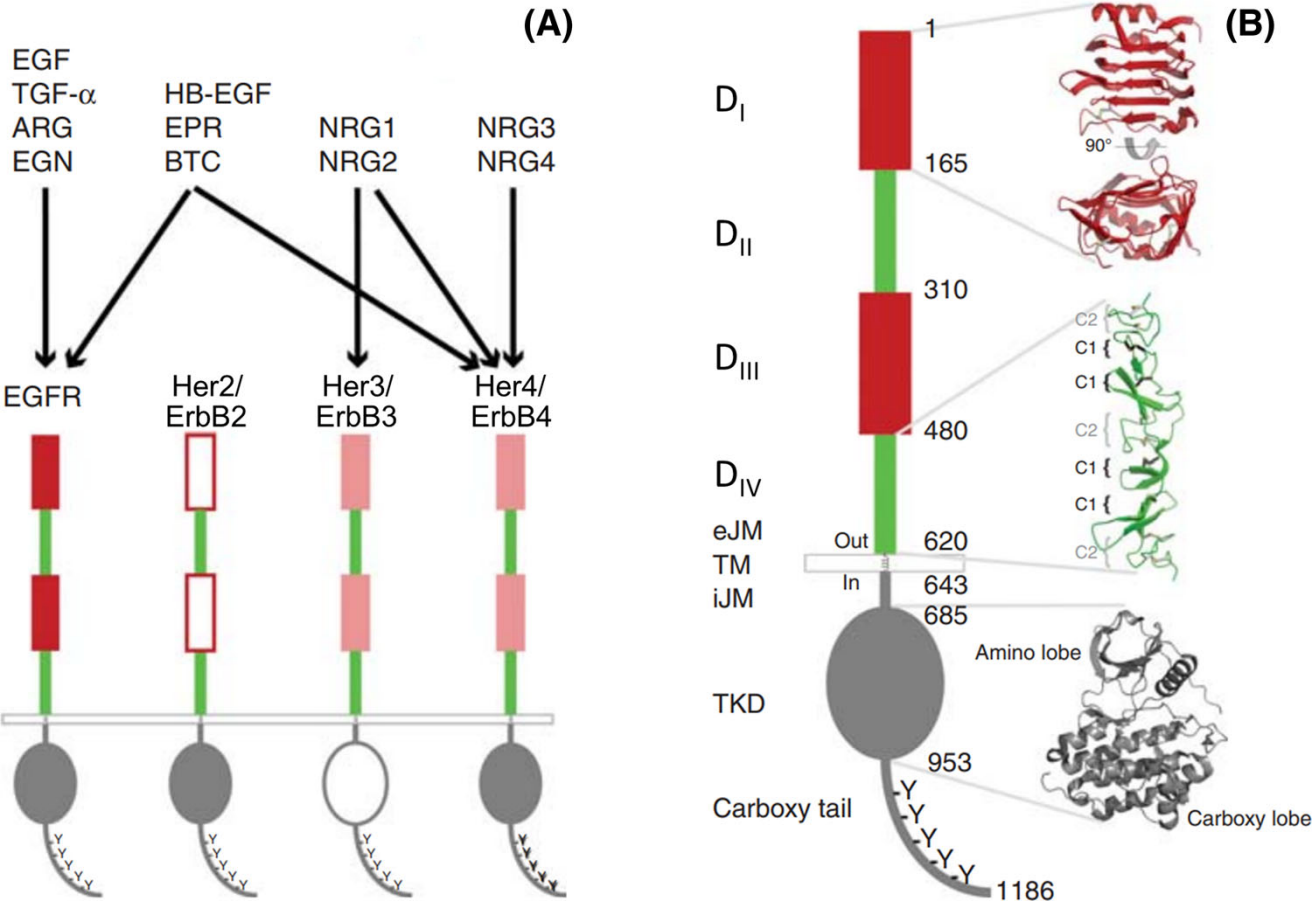
Schematic representation of EGFR/ErbB/Her family receptors.**^28^** **(A)** EGFR is one of four members of the EGFR/ErbB/Her family in humans. The other members are ErbB2/Her2, which is an orphan receptor without known soluble activating ligand; ErbB3/Her3 has a significantly impaired kinase domain^102^, ^114^; and ErbB4/Her4. EGFR binds and is activated by its cognate agonist growth factors: EGF itself, TGF‐α (transforming growth factor alpha), ARG (amphiregulin) and EGN (epigen). Bispecific ligands regulating both EGFR and ErbB4 are HB‐EGF (heparin‐binding EGF‐like growth factor), EPR (epiregulin), and BTC (betacellulin). Neuregulins (NRGs) 1 and 2 regulate ErbB3 and ErbB4, whereas NRG3 and NRG4 appear to be specific for ErbB4.^115^ **(B)** Domain composition of human EGFR. The extracellular region contains four domains. D_I_ and D_III_ are closely related in sequence, as are D_II_ and D_IV_. A short extracellular juxtamembrane (eJM) region separates the extracellular region from the transmembrane (TM) domain. Within the cell, a short intracellular juxtamembrane (iJM) region separates the tyrosine kinase domain (TKD) from the membrane. A representative EGFR tyrosine kinase domain (TKD) structure is shown. The TKD is followed by a carboxy‐terminal largely unstructured tail that contains at least five tyrosine autophosphorylation sites. Figure reprinted from Ref. (28). Copyright Cold Spring Harbor Laboratory Press

The EGFR family can be traced evolutionarily from one ligand and one receptor in *C. elegans*,^32^ through one receptor and at least four ligands in *Drosophila melanogaster*,^33^ to a family of four human epidermal growth factor receptors (Her1‐4) and at least 13 ligands.^34^ Among these ligands, seven can bind to EGFR (Figure 1A), four of these displaying intrinsic high affinity binding, namely epidermal growth factor (EGF), the first to be discovered (reviewed in Ref. 35), transforming growth factor alpha (TGF‐α), heparin‐binding EGF‐like growth factor (HB‐EGF), and betacellulin (BTC), whereas amphiregulin (AREG), epiregulin (EREG), and epigen (EPGN) are intrinsically low‐affinity ligands (reviewed in Ref. 34). Because EGFR in humans (aka Her1 and ErbB1) is at the heart of signals for growth and proliferation, it is frequently mutated and/or over‐expressed and thus hyper‐activated in human cancers,^36^ including non‐small cell lung cancer (NSCLC)^37^ and glioblastoma multiforme.^38^ For this reason, EGFR is an important target of anticancer therapy.^39^

## 3 EGFR MONOMER AND DIMERS ARE STRUCTURALLY WELL CHARACTERISED

Following its sequencing from cDNA clones derived from A431 epidermal carcinoma cells, where the *EGFR* gene is amplified 25‐fold, the complete 1210‐amino acid sequence of the EGFR became available.^40^ This includes a 24‐amino acid plasma membrane targeting peptide, the mature receptor deployed to the cell surface being composed of 1186 residues. EGFR displays a modular structure of interlinked domains (for a recent review see Ref. 3). As summarised in Figure 1B, starting from its extracellular N‐terminus, the EGFR is composed of a heavily glycosylated, ligand‐binding extracellular domain, built by four subdomains (D_I_–D_IV_), followed by a short extracellular juxtamembrane domain, a single transmembrane region, an intracellular juxtamembrane domain, a kinase domain, locus of the intrinsic protein tyrosine kinase activity of the receptor, and a long and largely unstructured regulatory C‐terminal tail. Ligand‐dependent EGFR autophosphorylation in the C‐terminal tail tyrosine residues is the crucial event that leads to the recruitment of intracellular effectors, their phosphorylation, and the ensuing signalling cascades that regulate cell function.^41^, ^42^ EGFR's vertebrate/human homologues (ErbB2/Her2, ErbB3/Her3, ErbB4/Her4) display the same modular structure (Figure 1A).

The activation of EGFR's catalytic activity depends on a well‐characterised transition from monomer to dimer. Revealed by X‐ray crystallography, key EGFR structures include the monomer kinase domain in complex with an inhibitor,^43^ a truncated back‐to‐back dimer of the extracellular domain that included D_I_‐D_III_ but missed D_IV_, and which was in complex with two EGF molecules,^44^ or two TGF‐α molecules,^45^ a so‐called ‘tethered’ ectodomain monomer,^46^ the catalytically active asymmetric tyrosine kinase dimer, made of an activator kinase which allosterically induces the active conformation of the ATP‐binding pocket of its receiver kinase partner,^47^ and the structure of an inactive symmetric head‐to‐head kinase domain dimer proposed to be autoinhibitory.^48^ NMR data revealed the dimer structures of C‐terminal and N‐terminal transmembrane dimers and of two juxtamembrane domain dimer arrangements catalysing the formation of the active asymmetric kinase dimer.^49^, ^50^, ^51^

**FIGURE 2:**
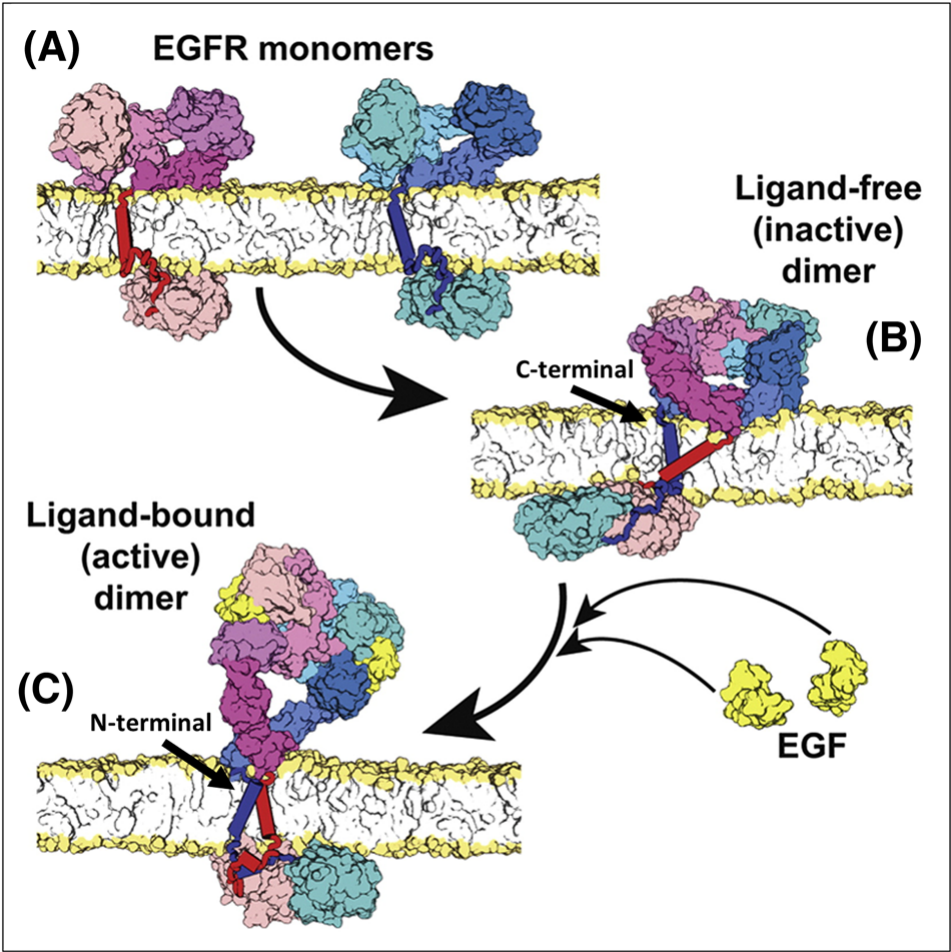
Near full‐length models of EGFR in a realistic membrane environment. **(A)** Model of the EGFR monomer; the simulation of the ectodomain was started from PDB entry 1NQL.^46^ The ectodomain is linked by a single helix embedded in embedded in a POPC/POPS membrane to the juxtamembrane and kinase domain in their inactive conformation.^48^, ^116^ **(B)** Model of the ligand‐free inactive dimer. The extracellular dimer was simulated starting from the crystal structure PDB entry 3NJP^87^ after removing the two bound ligands, which results in significant rearrangement of the c‐terminal portion of D_IV_, increasing their separation above the membrane. This favours a link with a C‐terminal transmembrane dimer and a membrane‐embedded juxtamembrane dimer connected via the extended juxtamembrane to the (inactive) symmetric kinase dimer.^48^ **(C)** Model of the ligand‐bound active dimer. The back‐to‐back extracellular dimer bound to two EGF molecules is linked to a N‐terminal transmembrane dimer, which induces the formation of an antiparallel dimer of the N‐terminal portion of the juxtamembrane domains that moves away from the plasma membrane and catalyses the formation of the asymmetric kinase dimer (PDB entry 2GS6).^47^ The latter is placed according to the orientation seen in the crystal structure PDB entry 3GOP.^117^ Reprinted from Ref. (52), Copyright (2013), with permission from Elsevier

All‐atom molecular dynamics (MD) simulations were instrumental to build from the above structures almost full‐length monomer and dimer models in the lipid bilayer that only lack the C‐terminal tail^52^ (Figure 2). The model of the monomer links the ectodomain via a single pass transmembrane helix with an inactive kinase domain that could interact with the plasma membrane by electrostatic interactions (Figure 2A). The extracellular portion of the ligand‐free, inactive ectodomain dimer was modelled by removing the two ligands bound to the progenitor back‐to‐back ectodomain dimer structure (Figure 2B). An important inference was the increased D_IV_‐D_IV_ separation at the C‐termini of the extracellular domains by the membrane, allowing linking to a C‐terminal transmembrane dimer that couples the extracellular dimer portion to an inactive symmetric head‐to‐head kinase dimer (Figure 2B). In the model of the active dimer (Figure 2C), the two ligand‐bound back‐to‐back ectodomain dimer seamlessly couple with a N‐terminal transmembrane dimer, which itself is structurally coupled with an antisymmetric helix dimer of the N‐terminal portion of the juxtamembrane domain known to favour the asymmetric kinase dimer. These models showed how ligand binding can control the shape of the extracellular domain dimer receptor, how the transmembrane and juxtamembrane segments alternate between two dimer forms, and how anionic lipids in the membrane are important to the regulation of the kinase domains.

## 4 FLUORESCENCE MICROSCOPY AS THE FOUNDATION OF THE LIGAND‐INDUCED DIMERISATION MODEL

Using image‐intensified video imaging, Schlessinger et al. pioneered fluorescence microscopy investigations of the aggregation state of EGFR bound to rhodamine derivatives of EGF ligand on the surface of 3T3 mouse fibroblasts.^53^ When cells were maintained at 4°C to inhibit receptor internalisation,^54^ results revealed that, as discerned by the diffraction limited resolution of optical microscopy (>250 nm), EGF‐bound receptors remain homogeneously distributed for at least 90 min. In contrast, when the temperature was raised to 37°C (physiological value), EGF‐bound receptors rapidly aggregated into patches larger than diffraction‐limited spots and were then internalised via receptor mediated endocytosis. The latter was proposed to be the mechanism of signal down‐regulation upon receptor degradation at lysosomes (reviewed in Ref. 55).

**FIGURE 3:**
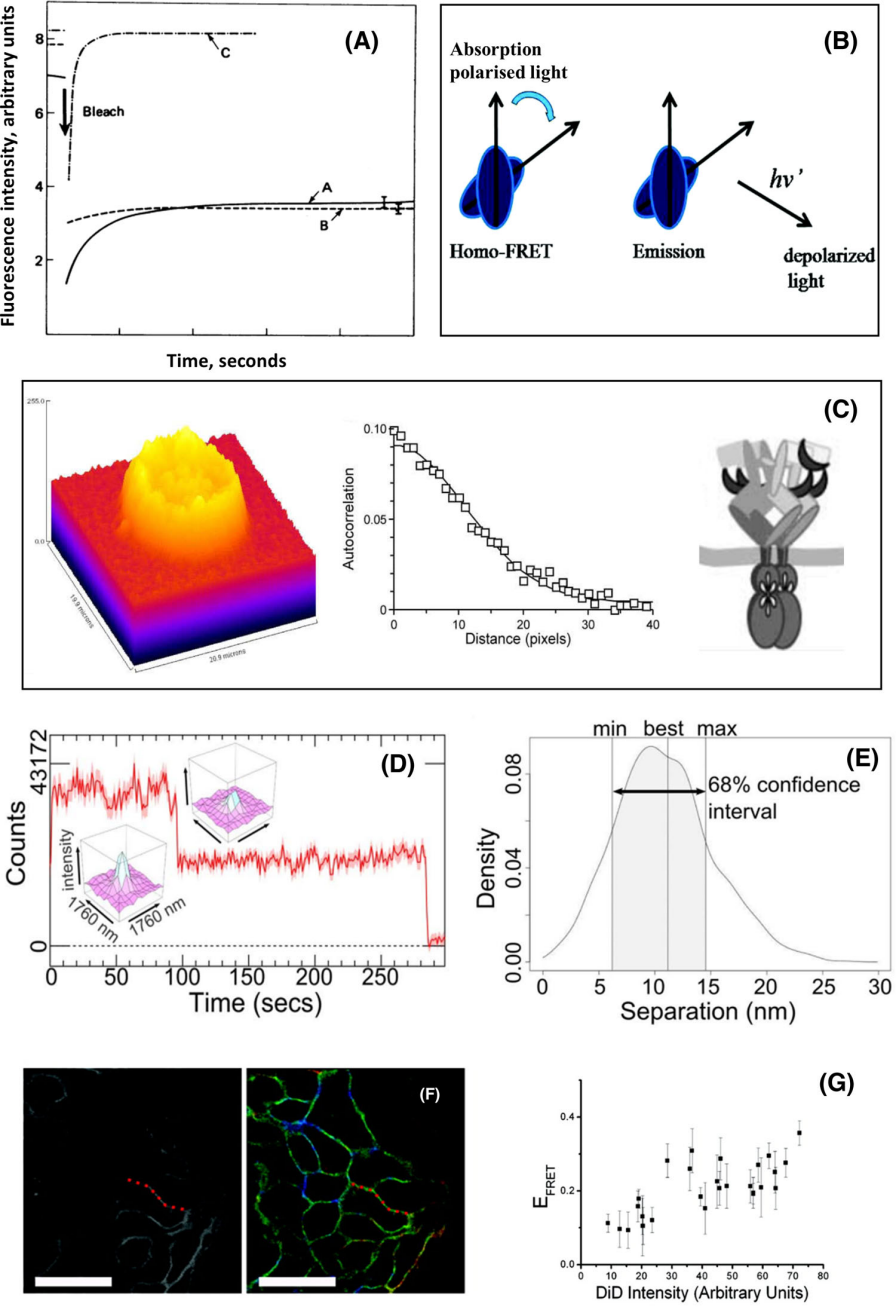
Example fluorescence microscopy methods to characterise state and conformation. (A) Photobleaching recovery curves of 3T3 cells at 23°C in the presence of 10 mM sodium azide to inhibit internalisation (curve A). Cells incubated for 20 min at 37°C in the presence of medium containing serum (curve B). For comparison control cells were labelled with the lipid probe DiI (3,3‐dioctadecylindocarbocyanine iodide) show fast recovery of ∼80% of the fluorescence that was bleached (curve C). From these data fractions of moving molecules and diffusion rates were calculated.^118^ **(B)** Cartoon illustrating how FRET depolarises the emission of the acceptor molecule. On absorption of vertically polarised light, the photonless transfer of excited state energy to the acceptor decouples the emission of the acceptor from the polarisation of the photon emitted by the donor. This is exploited in homo FRET as the degree of depolarisation scales with the number of transfer events.^66^ **(C)** (*left*) Confocal image of a BaF/3 cell expressing EGFR‐eGFP. The *z* axis represents the fluorescence intensity and the *x*, *y* axes the spatial coordinates.^64^ This image is an optical section taken near the cell equator and shows the membrane location of the EGFR‐eGFP and concentration fluctuation; (*middle*) spatial autocorrelation function: the average cluster density <*N*> ( = number of clusters/μm2) was determined from extrapolating the spatial autocorrelation function at zero lag (*g*(0)) using a Gaussian‐plus‐offset function as described by Petersen et al.^119^ (*right*) Model of the tetramer suggested by combining the image correlation and FRET data.^64^ (**D**) Example intensity bleaching time course of an image spot containing two molecules showing the change in the image after the first bleaching event.^5^ **(E)** An example of a seven‐parameter fit of the intensity and position of the two molecules and errors described by 1σ confidence intervals.^5^ **(F)** (*left*) Confocal image of acceptor intensity and (*right*) fluorescence lifetime image of the changes in the fluorescence lifetime of the FRET donor colour coded as a function of the degree of FRET efficiency.^72^ **(G)** Plot of the changes in FRET efficiency as a function of acceptor concentration derived from the area of membrane highlighted with red dots in (F). (F) and (G) Used with permission of American Society for Microbiology, from Ref. (72); permission conveyed through Copyright Clearance Center, Inc.

Schlessinger et al. also pioneered the use of FRAP to quantify EGFR mobility on previously bleached small cell surface regions (∼3 μm2)^53^ (Figure 3A). From the time course of the fluorescence intensity recovery in cells maintained at a temperature of 23°C, at which internalisation was still delayed, it was ascertained that 50–85% of the EGF‐bound cell surface receptor population were mobile on the plane of the membrane with a diffusion coefficient (*D*) of 3.5×10^−10^ cm^2^/s. When the temperature was increased to 37°C, >90% of receptors became incorporated in gross patches larger than diffraction‐limited spots and remained immobile (*D* < 10^−12^ cm^2^/s). Using chemicals to block oxidative phosphorylation, receptor aggregation was found not to require metabolic energy, seeming to depend on the rate of diffusion of the ligand occupied receptors.

Further work demonstrated that EGFR aggregation was required for receptor activation.^56^ This was followed by a series of elegant experiments on purified receptors, which suggested that signalling units were ligand‐bound EGFR dimers, and that these dimers become self‐phosphorylated *in trans*.^57^, ^58^ Based on this, the freely diffusing receptors detected by FRAP measurements were interpreted as inactive monomers that move laterally in the plasma membrane to interact with other receptors to form active signalling dimers, which then aggregate, become immobile and internalise. These early results set the foundations of the EGFR field, with the proposed ligand‐induced EGFR dimerisation model becoming the cornerstone of an allosteric signal transduction mechanism for EGFR, later described by X‐ray crystallography and NMR, as discussed above. While many aspects of this model were later supported by a wealth of data, including crystal structures of monomer and dimer EGFR fragments, despite its appealing simplicity, ligand‐induced dimerisation failed to explain some key principles of the functioning of EGFR assemblies in cells, as discussed below.

## 5 FROM DIMERS TO OLIGOMERS AND THE ELUCIDATION OF THEIR STRUCTURE

FRET microscopy was often used to measure inter‐molecular separations on cells expressing EGFR.^59^ Before crystal structures of EGFR dimers became available, FRET results were often interpreted in the context of ligand‐induced receptor dimerisation.^60^ A popular method to measure FRET in cells was to use fluorescent derivatives of mouse EGF, which, having no lysine residues, could be specifically labelled at its N‐terminus, and were found to bind the receptor without losing affinity.^61^ In 2002, crystallography revealed that the two EGF molecules in the 2:2 EGF/EGFR back‐to‐back dimer are bound at the flanks of the dimer with their N‐termini pointing away from each other, resulting in the separation between the N‐termini of bound EGF of ∼11 nm.^44^ This separation, which becomes 12.5 nm if one includes the typical size of popular organic probes,^62^ is outside the useful range that FRET can evaluate (<10 nm).^20^ FRET between donor/acceptor EGF‐derivatives could therefore not report dimer formation.

Given the evidence for significant FRET between EGFR‐bound probes, including high FRET efficiencies consistent with separations of <5 nm (see e.g. Refs. 63, 64, 65), the possibility that FRET might report receptor‐receptor interfaces in oligomers was considered. Because standard hetero FRET between spectroscopically different donor and acceptor probes is typically insensitive to stoichiometry, the possibility of EGFR oligomerisation was investigated using additional techniques.

One method employed was homo FRET, which involves the transfer of excited state energy between identical fluorophores.^66^ The degree of depolarisation depends on the number of homo FRET events, and thereby on the number of receptor‐bound probes transferring excited state energy to each other. Thus, by quantifying the anisotropy of the fluorescence emission due to energy transfer one can ascertain the size of stoichiometric oligomers (Figure 3B). Using cells transfected with EGFR that have been fused with monomeric green fluorescence protein (mGFP) at their C‐terminus, the presence of EGFR oligomers was detected by homo FRET.^67^

A breakthrough from Clayton et al. was to combine standard hetero FRET measurements with imaging correlation microscopy (ICM) (Figure 3C).^64^ While the latter can measure the number of molecules in clusters, FRET confirmed that the detected clusters were formed by close‐range receptor‐receptor interactions.^64^ Results suggested that tetramers were the most abundant EGF‐bound oligomer state. The small separation between ligands detected by FRET (∼4 nm) was interpreted as reporting side‐by‐side interactions between two EGF‐bound dimers forming the tetramer.

Needham et al. combined FRET with fluorophore localisation imaging with photobleaching (FLImP). FLImP is a single molecule localisation method that measures the separation between molecules emitting together in the same diffraction limited spot^5^, ^68^ (Figure 3D). This is achieved by exploiting single step photobleaching events to evaluate the positions of molecules in a diffraction limited spot with robust confidence intervals (Figure 3E), achieving resolutions <5 nm.^62^ For FLImP measurements we used a very stable, non‐sticky, hydrophilic CF640R‐EGF derivative.^69^, ^70^

**FIGURE 4:**
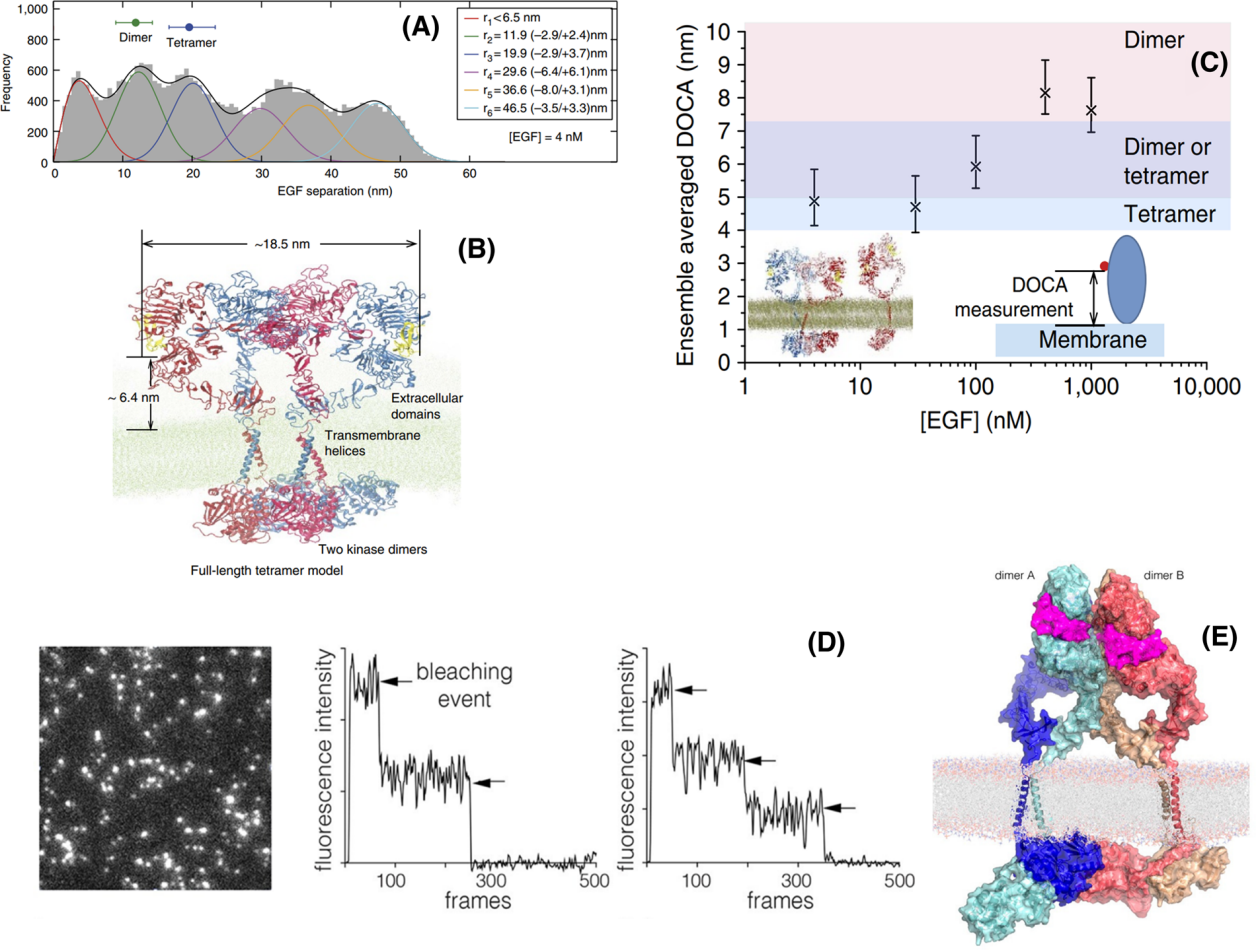
Structures of ligand‐bound oligomers. (A) FLImP distribution (grey) of CF640R fluorophore conjugated EGF on CHO cells (<10^5^ copies of wild‐type EGFR per cell) treated with 4 nM EGF. The peak positions (and error bars) marked above the plot reflect those expected for dimers (from crystal structures) and the tetramer from the MD simulation in B) after adding the size of the dye. The optimal number of peak components (colour lines) and the best‐fit (black line) were determined using a Bayesian information criterion and Bayesian parameter estimation.^62^ **(B)** The full‐length structural model of an EGFR tetramer as a dimer of active dimers assembled by the face‐to‐face interactions. The predicted separation between the N‐termini of the two EGF ligands and the average EGF‐membrane distance are marked. The oligomer can grow sideways via head‐to‐head interactions between dimers. **(C)** The distance of closest approach (DOCA) between EGFR‐bound EGF molecules and the membrane, derived from point‐to‐plain FRET measurements, for dimers and oligomers that form at different EGF concentrations (*x* axis).^62^ **(D)** (*left*) TIRF image of a Xenopus oocyte expressing EGFR, 2 min after addition of 15 nM EGF; (*middle and right*) representative photobleaching traces of the intensity in imaged spots. **(E)** A model for an EGFR tetramer, generated by connecting the model shown in (A) to the structure of the dimeric transmembrane helices (PDB code 2M20) and a chain of kinase domains (PDB codes: 2GS6 and 3GOP).^4^

FLImP measurements were accumulated in histograms to reveal the separations between ligand‐occupied receptors that are possible within oligomers distributed throughout flat regions of the basolateral cell membrane (Figure 4A). The results from these FLImP histograms validated long‐timescale all‐atom MD simulations independently conducted, which had revealed an architecture of EGF‐bound oligomers that was assembled from ligand less back‐to‐back dimers joined via a novel face‐to‐face interface^62^ (Figure 4B). These oligomers could reach sizes larger than tetramers, and FLImP results suggested they could reach up to decamer size. Interestingly, because the face‐to‐face interaction excludes ligand binding, these oligomers can only bind two ligands no matter their length, one bound to each of the two flanking protomers, thus displaying a 2:2N ligand/receptor binding ratio.

We combined the above FLImP measurements with point‐to‐plane FRET measurements of the separation between a receptor extracellular subdomain and the outer leaflet of the plasma lipid bilayer in which they are embedded.^71^, ^72^ The FRET measurements were undertaken using EGFR‐bound donor Alexa 488‐EGF probe, which bind D_III_ of the receptor, and the acceptor lipophilic dialkylcarbocyanine probe DiIC_18_(5) (DiD), which inserts itself in the plasma membrane with the chromophore lying on the outer leaflet^73^ (Figure 3F). For these FRET measurements, we used confocal microscopy and fluorescence lifetime imaging (for a recent review, see Ref. 74). From the efficiency of FRET measured as a function of acceptor concentration (Figure 3G), we calculated the mean separation between ligand‐bound receptors and the plasma membrane. From these results, we inferred that the ectodomain orientation of the ligand‐bound receptors FRET was consistent with the predictions from the MD simulations (Figure 4C).

Two months before our oligomer appeared, Huang et al. proposed an alternative structure derived from the combination of single molecule imaging and coarse grain simulations^4^ (Figure 4D). In common with our structure, this oligomer was also built from back‐to‐back dimer structures but rather than face‐to‐face interactions that exclude ligand binding, this other oligomer was assembled via extracellular side‐to‐side interactions (Figure 4E), thus lending support to a previous model proposed in Ref. (64). In this oligomer each constituent dimer can be bound to two ligand, the resulting ligand/receptor binding ratio is 2N:N.

It remains to be understood whether alternative oligomer structures carry out different functions in the cell, and whether thy coexist and/or cooperate, for example, to build larger architectures, such as the poorly characterised μm‐length EGFR clusters reported by STORM in normal and cancer cells.^6^ The existence of alternative structures is suggested by results from my lab that exploited smFRET methods^75^, ^76^, ^77^ analogous to those pioneered by Sako et al.^78^ to investigate on cells the conformational states of EGFR labelled using Cy3‐EGF and Cy5‐EGF derivatives as FRET pair. The results showed that FRET efficiency values clustered around two components. One displayed high FRET (separations <5 nm) and another low FRET (∼8 nm). This suggested two distinct states that could conceivably arise, for example, from two coexisting oligomer states.

## 6 ON THE ORIGIN OF HETEROGENEITY OF LIGAND BINDING

**FIGURE 5:**
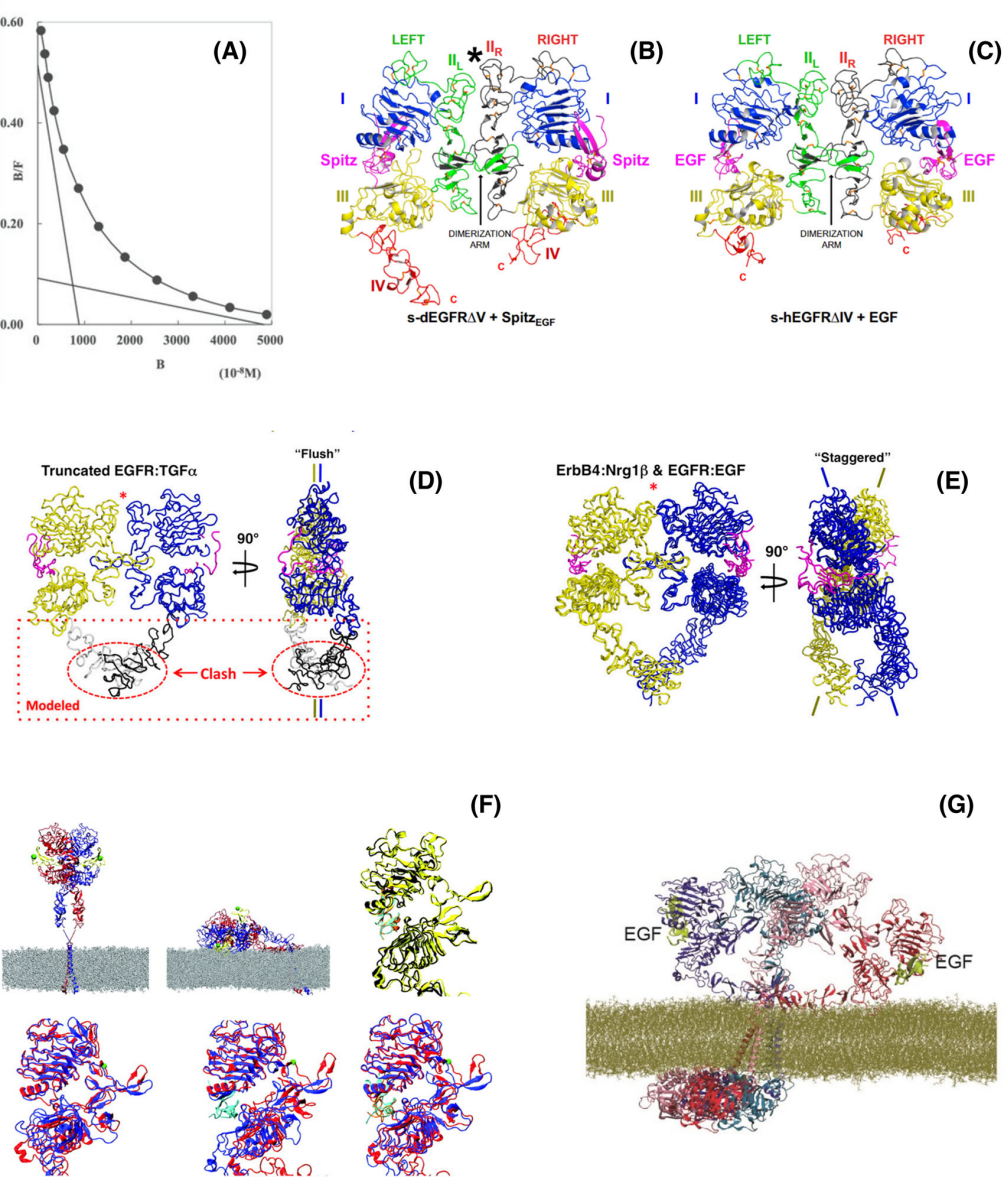
Heterogeneity of EGF binding to EGFR. **(A)** Quantitative binding experiments revealed a curvilinear dependence on the fraction of bound receptors versus the concentration of ligands yielding Scatchard plots like the one shown that can be fitted to two linear components.^120^ **(B)** The solubilised *Drosophila* EGFR extracellular domain (lacking D_V_) forms an asymmetric dimer when bound ligand (Spitz) [(Spitz_EGF_)_2_‐bound (s‐dEGFRΔV)_2_]. D_I_, D_III_ and D_IV_ are blue, yellow and red respectively. D_II_ is green in the left‐hand molecule (IIL) and dark grey in the right‐hand molecule (IIR). Bound Spitz is magenta. The dimerisation arm in D_II_ is labelled. An asterisk marks the amino‐terminal part of D_II_ where asymmetry is most evident.^85^ **(C)** Structure of the soluble truncated symmetric EGF‐induced dimer of the human EGFR extracellular region (s‐hEGFR) lacking D_IV_ (PDB code 1IVO),^44^ coloured as in (B). **(D)** Orthogonal views of worm diagrams of the truncated human EGFR dimer bound to TGFα (tEGFR:TGFα).^45^ The side‐on view (*right*) shows the flush conformation adopted by this truncated dimer. The predicted position of D_IV_ modelled on each subunit would predict a steric clash. **(E)** Orthogonal views of worm diagrams of dimers of soluble human ErbB4 extracellular domain bound to its ligand Nrg1β (s‐ErbB4:Nrg1β) and soluble human EGFR also including D_IV_ (s‐EGFR:EGF),^87^ following superposition of D_I_, D_II_ and D_III_. One receptor subunit is coloured yellow, the other blue; Nrg1β is coloured magenta.^86^ Superposition of a single receptor subunit of the tEGFR:TGFα dimer with a single subunit of either the sErbB4:Nrg1β or sEGFR:EGF dimers reveals the opposite ErbB subunits to differ by a scissor‐like rotation about the dimerisation arms. **(F)** (*top left*) Constrained by point‐to‐plane FRET data, the extended human receptor (hEGFR) ectodomain dimer with two bound ligands was modelled on crystallographic structures 1IVO^44^ and 1NQL^46^ and placed above modelled transmembrane helices in a POPC membrane. Receptor monomers are shown in red and blue ribbon representation, and both ligands are in yellow. Green spheres indicate the N termini of the ligands to which donor dyes are attached. (*top middle*) Endpoint of a MD simulation of a doubly liganded, tilted ectodomain human EGFR dimer, relaxed on the membrane.^89^ Also shown are overlays of the left and right subunits of receptor dimers using D_I_ as a reference for doubly liganded soluble human EGFR (1IVO). (*top right*) Simulation of unliganded hEGFR relaxed on the membrane. (*bottom left*) Simulation of singly liganded hEGFR relaxed on the membrane. (*bottom middle*) Simulation of doubly liganded human EGFR relaxed on the membrane. (*bottom right*) Unliganded soluble *Drosophila* EGFR (3I2T)^85^ shown in (B). Results suggested that by aligning on the plasma membrane the human EGFR dimer can recapitulate the asymmetry of the fly receptor.^72^ **(G)** The extracellular tetramer model in Figure 5B in a simulation of over 10 μs, in which the distance from one of the two bound EGF ligands to the membrane was particularly short, is consistent with FRET results used to constraint the simulations in (G). (A) From Ref. (120). Reprinted with permission from AAAS. (B), (C) Reprinted from Ref. (85), Copyright (2010), with permission from Elsevier. (D), (E) Reprinted from Ref. (86). (F) Used with permission of American Society for Microbiology, from Ref. (72).

Scatchard analysis of saturation binding of ^125^I‐labelled EGF to EGFR reliably yield curvilinear, concave up plots^79^ (Figure 5A). This suggests that besides intrinsic high and low affinity ligands, there is an additional receptor‐dependent source of heterogeneity of ligand binding. The Scatchard plots could be fitted by two linear components. The region of the Scatchard plot with the steep slope reported an estimated <10% of the total number of binding sites with an apparent ligand dissociation constant (*K_D_*) of <1 nM. The component with the shallow slope was consistent with a major class (>90%) of sites and a *K_D_* > 10 nM (reviewed in Ref. 80). The discovery that ligand binding to the sub‐class of high affinity sites is sufficient for activation of most canonical signalling pathways was facilitated by the observation that pre‐incubation with anti‐EGFR monoclonal antibody mAb 2E9 returned linear Scatchard plots that only display the steep slope.^81^ This is consistent with the blocking by mAb 2E9 of the binding of EGF to the low affinity sites. It was subsequently ascertained that low‐affinity binding is required for the activation of downstream signal effectors like the signal transducers and activators of transcription (Stats),^82^ the latter involved in many cellular responses including proliferation, migration and apoptosis (for a recent review, see Ref. 83).

Receptor‐dependent ligand binding heterogeneity could stem from negative cooperativity in an aggregating system or from two classes of independent receptor conformational states (reviewed in Ref. 84). There is evidence in the literature in support of both explanations. Consistent with negative cooperativity in an aggregating system, the X‐ray crystal structure of a truncated, solubilised ectodomain dimer of the *D. melanogaster* EGFR (s‐dEGFRΔV) showed a back‐to‐back structure in which the first ligand‐binding event induces the formation of an asymmetric dimer in which the unoccupied site is structurally restrained, thus reducing the affinity for the second ligand^85^ (Figure 5B). These results rationalised negative cooperativity in the invertebrate receptor.

Alternatively, in the human receptor results appeared to be best consistent with two independent receptor conformational states that display different affinity. This was suggested by the crystal structure of the ligand‐bound truncated, solubilised human ectodomain dimer (s‐hEGFRΔIV), which displayed a symmetric double‐ligated structure with two identical high‐affinity EGF binding sites^44^, ^45^ (Figure 5C). If both binding sites in the human dimer display equal affinity for ligand, given that the autoinhibitory tether interaction in the human ectodomain monomer would restrain the formation of the extended back‐to‐back dimer (Figure 2A), the early conclusion was that in vertebrates, monomers represent the lower affinity state and extended dimers the high affinity state (Figure 2B). However, subsequent experimental work and mathematical modelling argued that the high affinity class of EGFR in cells does not correspond to the extended configuration.^80^

Despite the lack of evidence for a single‐ligated asymmetric vertebrate EGFR dimer that would account for the observed ligand binding heterogeneity, single‐ligated dimers were identified in cells by co‐transfecting ligand binding‐deficient and kinase activation‐deficient EGFR mutants, which rescue phosphorylation when they interact together.^86^ The notion that these single‐ligated vertebrate dimers might display asymmetry, like in the fly homologue, was inferred by judicious structural analysis that compared the so‐called ‘flush’ dimer structure, displayed by the solubilised, truncated vertebrate ectodomain dimer in the absence of D_IV_ (Figure 5D),^86^ with the ‘staggered’ conformation found in the crystal structures of the full length solubilised ErbB4 ectodomain bound to two Neuregulin 1β molecules, and of the full length solubilised human EGFR ectodomain bound to two EGF molecules, where the ectodomain D_IV_ has not been truncated^87^ (Figure 5E). The flush and staggered conformations are related via a scissor‐like rotation about the dimerisation arms. A flush arrangement was observed in the asymmetric dimer of *Drosophila* sEGFR, in which only one receptor subunit has high affinity ligand bound, and a transition from flush to staggered being observed when a second ligand binds.^85^ Given this, it was reasoned that the flush conformation would be expected to occur in singly ligated ectodomain dimers of human EGFR that contain D_IV_. If this is the case, the requirement of a transition from flush to staggered would reduce the apparent affinity of the second site, resulting in negative cooperativity and a weaker receptor dimer, thus providing a rationale for the apparent negative cooperativity of ligand binding to human EGFR.

To investigate on cells the conformational states of EGFR that originate ligand binding heterogeneity, my lab exploited point‐to‐plane ensemble FRET methods to measure the mean vertical separation between donor Alexa 488‐EGF derivatives and the cell surface labelled with the membrane‐labelling acceptor chromophore DiI‐C_18_(3) (DiI).^88^ The latter were selected by pre‐incubating cells with mAb 2E9 to block low affinity sites. Results from these FRET experiments suggested that EGFR ectodomains displaying high affinity for EGF were tilted towards the plasma membrane.^63^ Interestingly, MD simulations suggested that tilted ectodomain orientations could afford close‐range interactions with the plasma membrane that may induce an asymmetric *Drosophila* EGFR‐like dimer in human EGFR^72^, ^89^ (Figure 5F).

Recent crystallography results suggested that when bound to an intrinsic low affinity ligand, like ERG, human EGFR forms a much weaker one‐ligand bound asymmetric back‐to‐back ‘flush’ dimer.^90^ Despite the weakness of the ERG‐bound asymmetric dimer interface, ERG‐bound EGFR asymmetric dimers elicit more sustained EGFR signalling than seen with EGF. Common glioblastoma multiforme mutations that occur in the extracellular domain have been shown to hijack the symmetric to asymmetric transition in the back‐to‐back dimer to prevent the receptor from discriminating between some activating ligands, namely between EGF and ERG.^91^

An alternative mechanism that could account for ligand binding heterogeneity was suggested by the oligomer structure assembled via face‐to‐face interactions between back‐to‐back dimers (Figure 4B). As the face‐to‐face interface includes the bulk of the ligand‐binding site, the face‐to‐face interface structurally restrains the binding of ligand. The latter must therefore outcompete this interface in order to bind. As a consequence of this, in these oligomers inner receptors bind ligand with lower affinity, thus providing a structural explanation of the origin of ligand‐binding heterogeneity in terms of negative cooperativity in an aggregating system.

Interestingly, all‐atom MD simulations of EGF‐bound oligomers suggested that the orientations of the two receptors at the ends of the 2:2N stoichiometry oligomers, which are the two receptors that would bind EGF with the highest affinity, can significantly tilt towards the plasma membrane (Figure 5G). This is consistent with point‐to‐plane ensemble FRET results in which mAb 2E9 was used to block EGF binding to low affinity sites.

## 7 DETERMINING THE NATURE OF THE LIGAND‐FREE INACTIVE STATE

The differences between the ability to derive crystal structures of strong single‐ligated fly EGFR asymmetric dimers but not of human EGFR underscored the importance that preformed (ligand‐free) intracellular dimers might have in stabilising ligand bound extracellular dimers of the human receptor, a notion supported by all atom MD simulations.^52^

Evidence of preformed non‐monomer complexes in humans was provided by fluorescence microscopy observations. Among the first cell‐based data were those derived ∼30 years ago via FRAP microscopy experiments on A431 cells, a cell line widely used for imaging because it overexpresses EGFR to ∼2×10^6^ copies/cell.^92^ FRAP revealed that EGFRs displaying high affinity for ligand were unable to move laterally in the plane of the plasma membrane, at least over a distance of a few hundred nanometres set by the diffraction limit of optical microscopy.^93^ Given that the high affinity EGFR subpopulation are those receptors that carry most of the signalling functions, if these receptors are immobile, the logical conclusion is that EGFR aggregation precedes the ligand stimulus.

Exploiting mAb 2E9 to block EGF binding to low affinity sites, in a pioneering FRET microscopy study Gadella and Jovin used fluorescein‐EGF and rhodamine‐EGF as a donor/acceptor pair to determine the efficiency of FRET from immobile high‐affinity sites in cells, imaged by epifluorescence microscopy.^61^ Evidence for FRET in these sires confirmed that high affinity receptors were pre‐aggregated at the nanoscale before they bound their cognate activating ligand.

Because of the high receptor surface density and potential for autocrine signalling ligand in A431 cells,^92^ further validation was sought out by fluorescence correlation spectroscopy (FCS)‐based techniques in combination with optical resolution microscopy methods, focusing on cells that express the receptor at low copy numbers. Models of choice included Chinese Hamster Ovary (CHO) cells and BaF3 cells, both of which do not express endogenous EGFR, its homologues and ligands.^94^ These cells can therefore be transfected to express physiologically relevant numbers of labelled EGFR copies in the absence of native unlabelled receptors. To ensure a degree of receptor labelling as close as possible to one‐to‐one, which is key to determine the number of receptors in clusters, cells were typically transfected with EGFR fusion constructs bearing fluorescence proteins, mostly enhanced green fluorescent protein (eGFP).^95^

Despite sample tuning efforts, evidence for constitutive receptor aggregation derived from FCS‐based results appeared to be inconsistent. For example, Nagy et al. examined in sequential confocal microscopy images of live CHO cells transfected with an eGFP‐EGFR fusion chimera the fluctuations of fluorescence intensities of single pixels.^96^ Results were analysed using the FCS‐related N&B technique proposed by Digman et al., reporting the mean molecular brightness and the number of molecules in ligand‐unstimulated clusters.^19^ The N&B results did not return evidence of pre‐aggregated receptor clusters on the cell surface at expression levels of 50,000–200,000 copies per cell. At the other extreme, using BaF/3 cells that stably expressed eGFP‐EGFR constructs at the level of ∼50,000 copies/cell, Clayton et al. reported using ICM that the bulk of unstimulated receptors exist on the surface as preformed dimers.^64^ Intermediate results were derived by combining FCS with fluorescence intensity distribution analysis (FIDA).^97^ These experiments were performed in live CHO cells transfected with an eGFP‐EGFR fusion construct and expressing ∼70,000 receptor copies per cell. The FIDA analysis suggested that ligand‐free receptors exist on the cell surface in an equilibrium involving 70% monomers and 30% of receptors distributed in clusters of two and more receptors. Similar results were obtained by Zanetti Domingues et al.^98^ We used ICM to reveal that in CHO cells that stably express EGFR to the tune of ∼<100,000 copies/cell, 25% of ligand‐free cell surface receptors were dimers and 35% higher order stoichiometric oligomers.

Other methods employed to investigate ligand‐free dimers were based on single particle tracking, imaging and localisation (for a recent review, see e.g. Ref. 99). Results from single molecule methods also appeared to be inconsistent. For example, Huang et al. imaged live Xenopus oocytes that express a very low copy number of an eGFP‐EGFR fusion construct and measured the number of single molecule fluorescence intensity photobleaching steps arising from single particle diffraction‐limited spots.^4^ Their results did not support the formation of EGFR aggregates in the absence of ligand stimulus. Chung et al. had earlier conjectured that perhaps a minimum number of EGFR per unit surface area might be required for unoccupied receptors to readily find each other and form a significant number of ligand‐free aggregates.^100^ In this work, EGFRs were labelled with quantum dots, which are semiconductor particles more resistant to photobleaching than organic dyes and FPs, thus allowing longer observations of the diffusion of individual receptor particles. Experiments were carried out in CHO‐K1 cell transfectants and stable cell lines displaying various degrees of EGFR expression. Assuming a Stokes‐Einstein‐like relationship *D* ∝ 1/*R* (in which *R* is the radius of the transmembrane domain),^101^ results suggested that most diffusing units are monomers and dimers, with the proportion of dimers being more abundant in the cell periphery, although the formation of higher oligomers was not excluded. Shortly afterwards, Low‐Nam et al. extracted kinetic parameters from single particle diffusion data in A431 cells using quantum dots to label EGFR.^1^ A three‐state hidden Markov model was used to identify transition rates between free, co‐confined and dimerised state. This, together with the localisation errors of <40 nm allowed by quantum dot brightness, revealed that transient, ligand‐free dimers readily form, with a kinetic stability that was in principle sufficient for ligand‐independent activation.^1^

## 8 AUTOINHIBITION MECHANISMS IN CONSTITUTIVE NON‐MONOMER STATES

The above results posed a crucial question: if EGFR forms ligand‐free aggregates, how can ligand binding activate a pre‐aggregated state? As discussed above, the mechanism suggested by all‐atom MD simulations is that ligand binding overcomes the autoinhibition imposed by the increased separation between the C‐termini of each D_IV_ that occurs in the ligand‐free back‐to‐back ectodomain dimer (Figure 2B). The increased proximity of the C‐termini of both D_IV_ in the 2:2 ligand‐bound dimer promotes an N‐terminal crossing transmembrane dimer, and thereby an N‐terminal juxtamembrane dimer that underpins the formation of the canonical asymmetric kinase dimer^102^ (Figure 2C). This mechanism of activation is also known as the rotation model.^103^

**FIGURE 6:**
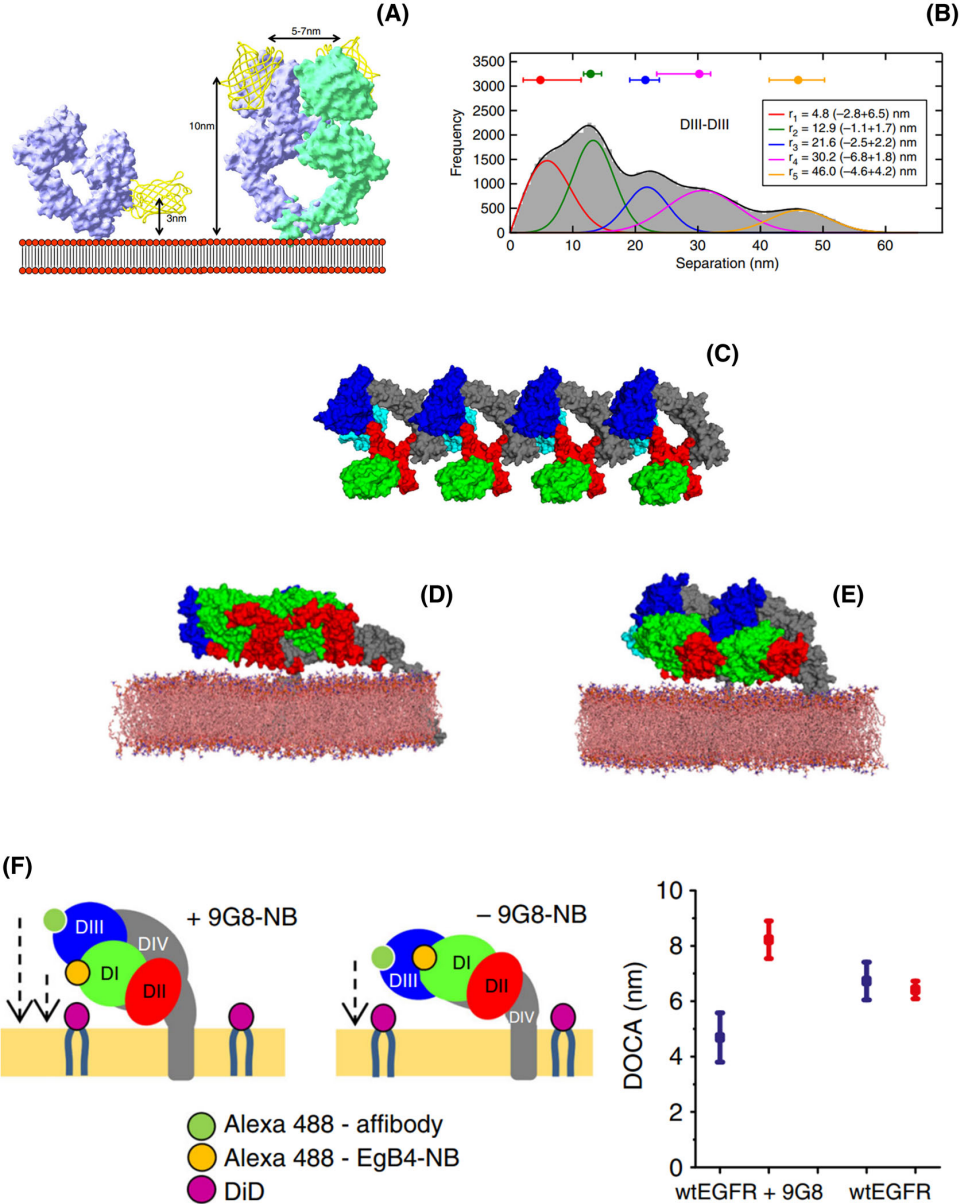
Autoinhibited conformations in dimers and oligomers. (A) Models of the YFP‐EGFR‐ectodomain on the cell surface membrane. The ectodomain (space filling model) is fused to YFP at its N‐terminus (FRET donor, yellow ribbon) in the tethered monomer (*left*) and untethered dimer (*right*) conformations. The membrane cartoon depicts the position of the rhodamine‐DHPE labels (red circles, FRET acceptor).^104^ Note the 3 nm separation between the YFP tag and the membrane in the tethered form that would be expected from high FRET efficiency) as compared to the untethered form (10 nm, low FRET efficiency). **(B)** FLImP distribution (grey) of D_III_–D_III_ separations between CF640R‐Affibody molecules bound to EGFR on CHO cells, compiled from FLImP measurements (CI ≤ 7 nm), decomposed into a sum of five components (coloured traces).^98^ The inset shows positions and error estimates. **(C)** An open‐ended oligomer model of 9G8‐bound EGFR extracellular domains in the inactive conformation built using the crystal contacts in the monomer structure in PDB ID 4KRP.^107^ **(D)** A simulation‐generated dimer structure of free EGFR extracellular domains and their TM domains in the lipid bilayer. The simulation was started from the crystal dimer of 9G8‐bound EGFR extracellular domains in the tethered conformation in which the two copies of the 9G8‐NB were removed from the simulation system. The images are based on the snapshot of the simulation at 20 μs. One of the two transmembrane helices is visible. **(E)** A simulation‐generated dimer structure of 9G8‐bound EGFR extracellular domains starting from a crystal dimer of 9G8‐bound EGFR extracellular domains in the tethered conformation. These images are based on the snapshot of the simulation at 20 μs. Invisible from this image are the TM helices embedded in the membrane. **(F)** (*left and middle*) Cartoons showing a side view of D_I_ and D_III_ separations from the membrane in head‐to‐head complexes in the presence and absence of bound 9G8‐NB; (*right*) FRET‐derived separations from the membrane‐DiI acceptor to D_I_ (Alexa 488‐EgB4‐NB, blue) or D_III_ (Alexa 488‐Affibody, red) donors. The FRET results were consistent with the predictions of the head‐to‐head dimer model.^98^ **(A)** Used with permission of IOP Publishing, from Ref. (104).

Experimental support for the extracellular portion of the autoinhibited, ligand‐free back‐to‐back dimer proposed by MD simulations shown in Figure 2B was derived by Kozer et al. using point‐to‐plane ensemble FRET measurements.^104^ FRET was measured between donor yellow fluorescent protein (YFP) fused to the N‐terminus of EGFR and the plasma membrane labelled with acceptor Rh‐DHPE (LissamineTM rhodamine B 1,2‐dihexadecanoyl‐sn‐glycero‐3‐phospho‐ethanol‐amine) (Figure 6A). The vertical separation between the N‐terminus of EGFR and the outer layer of the plasma membrane of BaF/3 cells, in which the combination of ICM and FRET had previously suggested the ligand‐free receptor mostly occupy the dimer state,^64^ was consistent with ligand‐free dimers standing proud from the cell surface, and thus consistent with the extended ligand‐free dimer configuration suggested by MD simulations. No crystal structure is available for the ligand‐free back‐to‐back dimer of flies and humans, but the ligand‐free *Caenorhabditis elegans* EGFR orthologue LET‐23 was crystallised displaying such a dimer.^105^

The rotational model of autoinhibition proposed by MD simulations turned out, however, to be too simple. Results from FCS and ICM experiments that suggested the existence of larger oligomers were confirmed at the nanoscale by Zanetti Domingues et al.^98^ We used FLImP to ascertain the architecture of ligand‐free complexes in CHO cells labelled with an antagonist anti‐EGFR Affibody probe that binds to D_III_ of the receptor's ectodomain.^106^ As shown in Figure 6B, the histogram of FLImP‐derived lateral separations between ectodomains in the oligomer, which was consistent with oligomers with a quasi‐linear shape, led to the prediction of a novel head‐to‐head ectodomain interface. Starting from lattice contacts of the tethered EGFR ectodomain co‐crystallised with a nanobody (EgA1) (PDB ID 4KRO)^107^ (Figure 6C), MD simulations revealed the structure of a ligand‐free head‐to‐head ectodomain monomer linked by interactions *in trans* between D_I_ and D_II_ (Figure 6D). This head‐to‐head dimer was also simulated in the presence of bound 9G8 nanobody (Figure 6E). As validation, point‐to‐plane ensemble FRET was used to measure the separations between ectodomain D_I_ and D_III_ and the plasma membrane using donor nanobody EgB4‐Alexa 488 to label D_I_ or donor Affibody‐Alexa 488 to label D_III_, with the plasma membrane labelled with acceptor DID. The FRET results reproduced the predictions of the MD simulations (Figure 6F). The autoinhibitory nature of the head‐to‐head dimer resides in the separation imposed by this interface between transmembrane domains (Figure 6D), which prevents the formation of the catalytically active asymmetric kinase dimer. This work also showed that EGFR lung cancer mutations overcome the autoinhibitory block of the head‐to‐head interface, thereby becoming constitutively activated losing the regulation provided by the ligand.

## 9 FUTURE PERSPECTIVES

Despite the vast multidisciplinary knowledge accumulated on the structure of EGFR, critical questions remain unanswered. We need to understand the different architectures that can be assembled by EGFR at different functional regions of the plasma membrane and different intracellular cell locations. This should allow us to elucidate the mechanisms EGFR uses to govern the timing and composition of the supra‐molecular complexes assembled to elicit specific early and late cellular outcomes. We also need to understand the changes induced by co‐receptors, including its family homologues HER2/ErbB2 and HER3/ErbB3, together crucial in breast cancer, and elucidate how these interactions modulate structural states of normal EGFR and of cancer mutations, together with the associated changes to their signalling output. These are huge tasks, for which fortunately now have at our disposal tools of exquisite resolution. These include MINFLUX,^108^ with a nominal resolution of 2–4 nm in 3D, a technique which has already made inroads in our understanding of, for example, the organisation of mitochondrial MICOS proteins,^109^ and the unimpeded walk of kinesin‐1^110^ (for a recent review see Ref. 111). Another technique revealing unprecedented information of cellular structural biology is correlative light and electron microscopy (CLEM). Combined with cryo‐fixation, a methodology suitable to preserve ultrastructure at nm resolution, CLEM can reveal the structure of specific proteins, labelled by fluorescence, in the mix of many other surrounding proteins in the cell (for a recent review see Ref. 112). Cryo‐CLEM could in principle also reduce the current dependence on MD simulations to provide structural context (for a recent review see Ref. 113). By combining the above with the advances brought by artificial intelligence methods, it feels safe to predict a bright future for our understanding of EGFR cellular structural biology. Critically, knowing how these states become dysregulated by cancer‐driving EGFR mutations is paramount to rationally design new anticancer drugs and to predict their action in different patient cohorts. The expectation is that this should feed into better and more efficacious treatments, hopefully enabling a step change in the development of anticancer treatments.
